# The effect of step count on pain and quality of recovery after cholecystectomy: a prospective observational cohort study

**DOI:** 10.3389/fsurg.2026.1726792

**Published:** 2026-01-27

**Authors:** Busra Cetin, Hatice Yuceler Kacmaz, Fatih Dal

**Affiliations:** 1 Department of Fundamentals of Nursing, Faculty of Health Sciences, Erciyes University, Kayseri, Türkiye; 2 Department of Surgical Nursing, Faculty of Health Sciences, Erciyes University, Kayseri, Türkiye; 3 Department of General Surgery, Faculty of Medicine, Erciyes University, Kayseri, Türkiye

**Keywords:** cholecystectomy, pain, pedometer, quality of recovery, step count

## Abstract

**Aim:**

The study was conducted to determine the effect of postoperative step count on pain and quality of recovery in patients undergoing cholecystectomy.

**Method:**

This prospective observational cohort study was conducted with 136 patients who underwent cholecystectomy at a university hospital between October 2023 and July 2024. Preoperatively, patients' information was obtained, and a 6-minute walking test was performed. Postoperatively, the number of step counts recorded by the pedometer and pain levels assessed by the Visual Analog Scale were monitored for three days. On postoperative day three, the patients were administered Quality of Recovery-15 (QoR-15). Data were analyzed using descriptive statistics, Pearson correlation analysis, and multivariable linear regression.

**Results:**

After cholecystectomy, the median postoperative step count was 245 on day 1, 719.50 on day 2, and 983.50 on day 3. Mean pain scores were 7.20 ± 1.10 on postoperative day 1, 5.26 ± 1.29 on day 2, and 3.76 ± 1.42 on day 3. On postoperative day 3, the mean total QoR-15 score was 124.75 ± 18.71. Postoperative step counts were negatively correlated with pain scores and positively correlated with total and subscale QoR-15 scores. In multivariable linear regression analysis, postoperative recovery quality was independently associated with surgical approach, step count on postoperative day 3, and pain level on postoperative day 3. Higher step counts on postoperative day 3 were associated with higher QoR-15 scores, whereas higher pain levels were associated with lower QoR-15 scores.

**Conclusion:**

The findings indicate that increased postoperative physical activity is associated with reduced pain and improved quality of recovery after cholecystectomy, highlighting the importance of maintaining postoperative mobility as part of perioperative care.

## 1 Introduction

Cholecystectomy, the gold standard method in the treatment of gallbladder diseases, is among the most commonly performed surgical procedures worldwide, with more than 750,000 procedures performed annually (1–3). Although surgery is an important treatment method in ensuring that the patient regains his/her health, it also brings many undesirable events. Complications such as surgical site infections, bleeding, bile leakage, biliary tract injuries, deep vein thrombosis, pulmonary embolism, and postcholecystectomy syndrome can be seen in patients after cholecystectomy. Therefore, follow-up of the recovery process is essential in post-cholecystectomy care (4, 5). While postoperative recovery has traditionally been evaluated using outcomes such as complications, mortality, and length of hospital stay, the quality of recovery has gained increasing attention in recent years as a patient-reported outcome (6, 7). The quality of recovery, which reflects the restoration of preoperative physical independence, enables patients to actively participate in their care by expressing their perceptions of health status, outcomes, and quality of life (8–10). Improved quality of recovery has been associated with shorter hospital stays, earlier return to daily activities, and fewer complications (6, 11).

Previous studies have shown that multiple factors influence the quality of recovery, including pain, early mobilization, physical mobility, nutrition, anxiety, and psychosocial support (12, 13). Among these, physical mobility plays a central role in the surgical recovery process by preventing complications and promoting rapid functional recovery (14). Prolonged postoperative immobilization adversely affects multiple body systems, leading to muscle weakness, impaired pulmonary function, and an increased risk of thromboembolic and cardiac complications (14, 15). Early and regular postoperative mobilization has been shown to reduce the length of hospital stay, accelerate recovery, facilitate discharge, and improve patient satisfaction, thereby enhancing overall quality of recovery and reducing healthcare costs (5, 13–15)*.*

When the literature is examined, it is seen that studies focus on the effect of early mobilization on pain and/or quality of recovery (16, 17), and there are limited studies evaluating the sustainability of physical mobility of patients after early mobilization (13, 18–20). In addition, there are no studies evaluating physical mobility in patients undergoing cholecystectomy and revealing its effect on pain and quality of recovery.

When the literature is examined, most studies have focused on the effects of early mobilization on postoperative pain and/or quality of recovery (16, 17). However, evidence regarding the sustainability of postoperative physical mobility remains limited (13, 18–20). Moreover, to the best of our knowledge, there are no studies that objectively evaluate postoperative physical mobility in patients undergoing cholecystectomy and examine its relationship with pain and quality of recovery. Therefore, this study aimed to assess postoperative physical mobility using step count measurements objectively and to explore its association with postoperative pain and quality of recovery in patients undergoing cholecystectomy.

## 2 Methods

### 2.1 Study design

The research is a prospective observational cohort study to determine the effect of postoperative step count levels on pain and quality of recovery in cholecystectomy patients.

#### 2.1.1 Research questions

What are the postoperative step count patterns of patients during the first three days following cholecystectomy?
Is postoperative step count associated with pain intensity and quality of recovery across the first three postoperative days following cholecystectomy?
Is postoperative step count independently associated with quality of recovery after cholecystectomy?

### 2.2 The population and sample

The study was conducted between October 2023 and July 2024 in the general surgery ward of a 1,200-bed tertiary university hospital in Türkiye. The ward consists of two departments and has a total capacity of 70 beds. In the ward, patients are usually mobilized 8–10 h after cholecystectomy surgery. The forms used to determine the pain levels of the patients in the ward are filled out by the nurses after the surgical procedure.

#### 2.2.1 Power analysis

**Figure 1:**
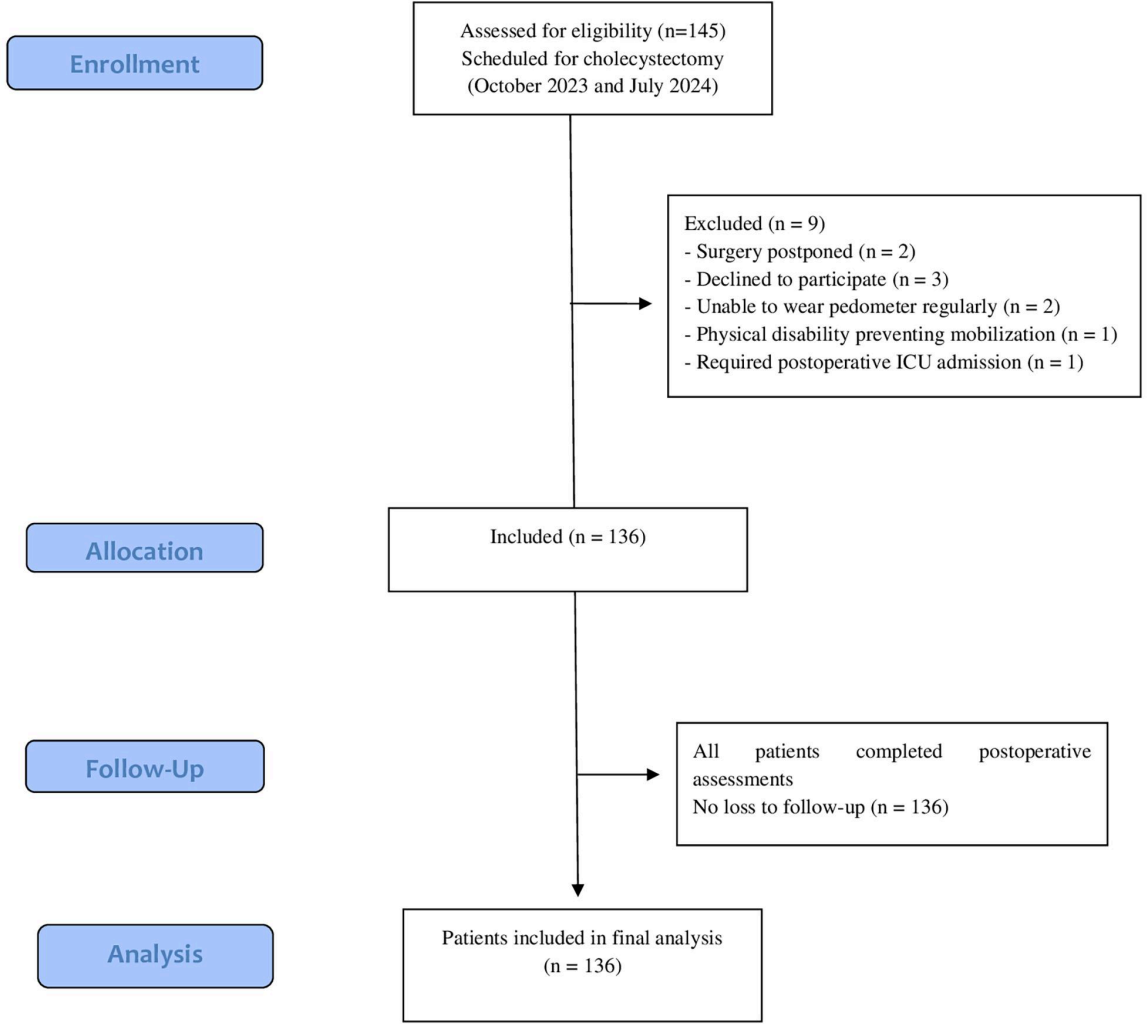
Flowchart of participant recruitment.

To determine the sample size of this study, a reference study (20) in which the number of step counts and quality of recovery were evaluated together was used. When calculating the sample size, the effect size was taken as 0.31, *α* = 0.05, *β* = 0.98 and the sample size was calculated as = 131. Considering that there would be data loss during the research, it was decided to include 145 people, 10% more than the sample size, in the study. Of the 145 patients who underwent cholecystectomy between October 2023 and July 2024; two patients had postponed surgery, one patient was admitted to intensive care, three patients did not accept the study, two patients could not wear the pedometer regularly, one patient had a physical disability, and the research was completed with 136 people (Figure 1).

The study included patients who were over 18 years of age, had no communication problems, had cholecystectomy, underwent elective surgery, and were in any of the ASA I, ASA II, or ASA III groups according to the American Society of Anesthesiologists (ASA) Physical Status Classification System, volunteered to participate in the study. The study exclusion criteria who refused to wear a pedometer, 6 min walk test contraindicated (unstable angina pectoris within the last month, systolic blood pressure above 180 mm Hg, diastolic blood pressure above 100 mm Hg, resting heart rate above 120), using an assistive walking device such as cane, walker, etc. while walking, individuals with physical mobility disabilities were not included in the study. Patients whose surgery was postponed after inclusion, who needed intensive care in the postoperative period, who could not be reached within three days after surgery, and who had a walking distance below the average walking distance according to the 6-minute walk test (20–50 years = 590 m, 60–70 years = 570 m) and who did not wear a pedometer for 3 days were also excluded from the study.

Minimum walking distance thresholds based on age-specific reference values were applied to ensure that participants had sufficient baseline functional capacity to perform postoperative ambulation and to allow reliable assessment of step counts. These criteria were used to minimize measurement bias related to severe preoperative mobility limitations.

### 2.3 Data collection

The first interview with the patients was conducted in the preoperative period when the patients were admitted to the clinic (Pre-1). The information of the patients who met the inclusion criteria was obtained (Table 1) and a 6-min walk test was performed. In the study, the 6-minute walking test was to obtain information about the patient's physical mobility and functional capacity in the preoperative period (21). The distance that the patients could walk for 6 min was measured in a 30-meter-long straight corridor in the ward. The study was terminated with patients aged 20–50 years walking 590 meters and patients aged 50–70 years walking less than 570 meters.

**Table 1:** Characteristics of patients undergoing cholecystectomy (*n* = 136).

Characteristics	*n*	%
Medical diagnosis		
Cholecystitis	24	17.6
Cholelithiasis	112	82.4
Age (x¯ ± SS)	50.76 ± 14.67	
Gender		
Male	60	44.1
Female	76	55.9
Marital status		
Married	114	83.8
Single	22	16.2
Education		
Primary education (5 years)	62	45.6
Middle-High degree (6–11 years)	41	30.1
Undergraduate degree (>12 years)	33	24.3
Work		
Working	54	39.7
Not working	82	60.3
BMI		
18–24.9	36	26.5
25–29.9	54	39.7
≥30	46	33.8
Smoking		
Yes	38	27.9
No	98	72.1
Chronic diseases		
No	50	36.8
Yes	86	63.2
HT	28	32.6
DM	22	25.6
Chronic heart disease	18	20.9
Other	18	20.9
Surgery history		
Yes	88	64.7
No	48	35.3
Surgical approach		
Open	10	7.4
Laparoscopic	126	92.6
ASA		
1	118	86.8
2	18	13.2

BMI, body mass index; ASA, American Society of Anesthesiologists Classification.

In the postoperative period, four interviews were conducted before mobilization, on the first day (24th hour), the second day (48th hour), and the third postoperative day (72nd hour). In the first interview after the surgical procedure, before the patient was mobilized (Post-1), pain assessments were made according to the VAS before the first mobilization, and the patients were given a pedometer. Patients were informed about the pedometer and reminded not to remove it. Eight to ten hours following the surgery, the patients were initially mobilized with the physicians and nurses. Interviews were continued with the patients on the first postoperative day (Post-2), second postoperative day (Post-3), and third postoperative day (Post-4). In this process, pain assessments were made according to VAS, and the number of step counts on the pedometer was checked and recorded in the patient follow-up chart. After each recording, the number of step counts on the pedometer was reset to measure the daily number of step counts after surgery. In addition, the quality of recovery-15 (QoR-15) scale was completed to assess the quality of recovery at the Post-4th interview. In the Post-3rd and Post-4th interviews, interviews were conducted in the patient's hospital room if the patient remained hospitalized in the clinic, and by telephone if the patient was discharged.

#### 2.3.1 Data collection tools

##### 2.3.1.1 Visual analog scale (VAS)

Developed by Price et al., VAS is used to determine the severity of pain in the patient. VAS is a scale with high reliability and sensitivity that is widely used in clinics for its quick and easy applicability. This scale consists of a 10 cm line with values ranging from 0 to 10. The beginning of the line (number 0) indicates “no pain”, while the end of the line (number 10) indicates “the most severe pain” (22).

##### 2.3.1.2 Quality of recovery-15 (QoR-15) scale

This is a shortened version of the QoR-40 scale, developed by Myles et al. The scale was adapted into Turkish by Kara et al. The QoR-15 scale is a unidimensional scale measuring the quality of recovery. With this scale, the quality of recovery is assessed in five domains. These domains are physical comfort, pain, physical independence, psychological support, and emotional state. The scale consists of two sections, A and B, with 10 questions in section A and 5 questions in section B, totaling 15 questions. Questions 1, 2, 3, 4, and 13 are in the “physical comfort” subscale; questions 5 and 8 are in the “physical independence” subscale; questions 9, 10, 14, and 15 are in the “emotions” subscale; questions 6 and 7 are in the “psychological support” subscale; and questions 11 and 12 are in the “pain” subscale. In section A, each question is “never (0)” or “always (10)”, while the questions in section B indicate “never (10)” represents “always (0)”. A minimum score of 0 and a maximum score of 150 can be obtained from the QoR-15 scale. As you move from 0 to 150 points, the quality of recovery increases (9, 23).

##### 2.3.1.3 Physical mobility

Physical mobility in the post-operative period was monitored through the number of step counts of the patients, which was measured using a pedometer. The pedometer (PlusMed Step4 Health Step) features include a pedometer, date and time settings, a large screen, a belt clip, a three-dimensional sensor, six walking modes, time, walking distance, calories burned, walking speed, and activity time displayed on the screen. The battery-powered pedometer was preferred as a data collection tool in the study because it does not create a harmful factor such as radiation to the individual while working. It was worn on the waist of all individuals participating in the study. In addition, the cover on the pedometer allows it to be smaller and keeps button pressing under control.

### 2.4 Data analysis

The data obtained from the study were analyzed in the IBM SPSS Statistics 24.0 (IBM Corp., Armonk, New York, USA) package program. Descriptive statistics number (*n*), percentage (%), mean and standard deviation (SD), median, minimum (min), and maximum (max) were used to evaluate the data related to patient characteristics. The conformity of the data to normal distribution was evaluated by skewness and kurtosis tests. The Pearson correlation test was used to determine the relationship between the number of step counts and the VAS scores and QoR-15 total score. Multivariable linear regression analysis was conducted to identify factors independently associated with QoR-15 total score. The results were evaluated using a 95% confidence interval and a significance level of *p* < 0.05.

## 3 Results

The mean age of the participants was 50.76 ± 14.47 years, 39.7% were between 40 and 59 years, 55.9% were female, 45.6% were primary school graduates, and 83.8% were married. It was observed that 82.4% of the individuals had undergone cholecystectomy with cholelithiasis, 39.7% had a body mass index between 25 and 29.9, and 60.3% had no working life (Table 1).

The mean of the walking test performed in the preoperative period was 644.69 ± 39.09 and the median number of step counts on the postoperative days was 245 on the first day, 719.5 on the second day, and 983.5 on the third day, respectively (Table 2).

**Table 2:** Step counts of patients undergoing cholecystectomy (*n* = 136).

Step counts	$\bar{X}$	SS	Median	Min–max
Walking test	644.69	39.09	635.0	580–760
Post op 1	216.57	139.83	245.0	53–950
Post op 2	693.93	336.83	719.5	126–1,780
Post op 3	949.13	394.30	983.5	162–1,816

The mean pain scores on the postoperative days were 7.20 ± 1.10 on the first day, 5.26 ± 1.29 on the second day, and 3.76 ± 1.42 on the third day, respectively (Table 3).

**Table 3:** Postoperative pain scores of patients udergoing cholecystectomy (*n* = 136).

VAS Score	$\bar{X}$	SS	Median	Min–max
Post op-1 (8th hour)	7.20	1.10	7.0	5–10
Post op-2	5.26	1.29	5.0	3–8
Post op-3	3.76	1.42	4.0	2–7

VAS, visual anolog scale.

The mean postoperative QoR-15 total score of individuals who underwent cholecystectomy was 124.75 ± 18.71 and the mean scores of the subscales of the scale were 40.94 ± 6.33 in the physical comfort, 33.75 ± 7.19 in the emotion, 14.30 ± 3.89 in the physical independence, 19.58 ± 1.27 in the psychological support, and 16.16 ± 3.03 in the pain, respectively (Table 4).

**Table 4:** Postoperative QoR-15 scores of patients undergoing cholecystectomy (*n* = 136).

QoR-15 score	$\bar{X}$	SS	Median	Min–max
Comfort	40.94	6.33	42.0	26–50
Emotion	33.75	7.19	36.5	12–40
Independence	14.30	3.89	15.0	4–19
Support	19.58	1.27	20.0	13–20
Pain	16.16	3.03	17.0	6–19
Total	124.75	18.71	128.5	74–147

QoR-15, quality of recovery 15 scale.

There was a low negative correlation (*r* = 0.142–0.176) between the number of step counts on the postoperative first day and pain scores (*p* < 0.05). There was a moderate (0.482–0.608) negative correlation between the number of step counts on postoperative second day and pain scores and a low (*r* = 0.384) and moderate (*r* = 0.488–0.677) negative correlation between the number of step counts on postoperative third day and pain scores (*p* < 0.01). As the number of postoperative step counts of individuals who underwent cholecystectomy increased, their pain scores decreased (Table 5).

**Table 5:** Correlation of step counts and pain and QoR-15 scale of patients undergoing cholecystectomy.

Step counts	VAS Post-op 1	VAS Post-op 2	VAS Post-op 3	Comfort	Emotion	Independence	Support	Pain	QoR-15 Total
Post-op 1	−0.176*	−0.170*	−0.142*	0.230**	0.150	0.248**	0.132	0.269**	0.240**
Post-op 2	−0.482**	−0.608**	−0.615**	0.517**	0.493**	0.508**	0.073	0.577**	0.530**
Post-op 3	−0.384**	−0.488**	0.677**	0.655**	0.511**	0.561**	0.183*	0.671**	0.656**

VAS, visual anolog scale, QoR 15, quality of recovery 15 scale.

* *p* < 0.05.

** *p* < 0.01.

Multivariable linear regression analysis was performed, adjusting for age, gender, chronic disease, smoking status, surgical approach, postoperative pain scores, and postoperative step counts. The analysis showed that the quality of recovery was independently associated with surgical approach, step count on postoperative day 3, and pain level on postoperative day 3. Higher step counts on postoperative day 3 were associated with higher QoR-15 scores (B = 0.015, *β* = 0.311, *p* = 0.001), while higher pain levels on postoperative day 3 were associated with lower QoR-15 scores (B = −6.164, *β* = −0.468, *p* < 0.001). The model explained 53.1% of the variance in QoR-15 scores (adjusted R^2^ = 0.531) (Table 6).

**Table 6:** Multivariable linear regression analysis of the postoperative QoR-15 scale.

Model		Unstandardized coefficients		Standardized coefficients	*t*	*p*
		B	Std. Error	Beta		
1	(Constant)	97.057	13.848		7.009	.000
	Age	.095	.103	.074	.918	.361
	Gender	.107	3.045	.003	.035	.972
	Chronic disease	−1.687	2.795	−.044	−.604	.547
	Surgical approach	15.705	4.687	.220	3.351	.001
	Smoking	−4.176	2.926	−.100	−1.427	.156
	Step counts (Post-op 1)	.005	.009	.040	.602	.548
	Step counts (Post-op 2)	.004	.005	.066	.667	.506
	Step counts (Post-op 3)	.015	.004	.325	3.477	.001
	VAS (Post-op 1)	1.952	1.766	.116	1.105	.271
	VAS (Post-op 2)	−.017	1.776	−.001	−.010	.992
	VAS (Post-op 3)	−5.594	1.771	−.425	−3.158	.002

Dependent variable: QoR-15 scale.

VAS, visual anolog scale, QoR 15, quality of recovery 15 scale.

## 4 Discussion

To our knowledge, this is the first study to monitor postoperative physical mobility after cholecystectomy using a pedometer objectively and to examine its association with pain and quality of recovery. In this study, postoperative step counts gradually increased over the first three days following surgery, with median values of 245 steps on postoperative day 1, 719.50 steps on day 2, and 983.50 steps on day 3. Since there are no studies in the literature evaluating the step counts of cholecystectomy patients, the results related to step counts are discussed with the findings of abdominal surgery patients. Massouh et al. conducted a prospective cohort study to examine the effect of physical mobility, as measured by an activity tracker, on the quality of recovery in individuals (*n* = 200) after cesarean section. It was reported that the number of step counts 24 h after a cesarean section should be at least 287 to affect recovery positively (20). This step count is similar to the step counts on the first day of our study. In a randomized controlled study conducted by Wilnerzon Thörn et al., the effect of the rapid mobilization process (initiated 30 min after surgery) on the physical activity process was evaluated in patients (*n* = 144) who underwent laparoscopic and robotic colorectal surgery. The median number of step counts of the patients in the control group was 404 on the first day, 519 on the second day, and 762 on the third day (24). In a randomized controlled pilot study, Wiesenberger et al. examined the effect of motivational interviewing on postoperative mobilization in patients undergoing elective colorectal surgery. The authors reported that median step counts during the first three postoperative days were consistently higher in the intervention group compared with the control group, with values of 646, 1,882, and 1,783 steps vs. 336, 1,030, and 1,030 steps, respectively (25). Although the findings of the present study are broadly consistent with those reported in the literature, differences in study populations, surgical approaches, and measurement instruments should be considered when interpreting these results.

In our study, the postoperative pain levels of patients who underwent cholecystectomy were 7.20 ± 1.10, 5.26 ± 1.29, and 3.76 ± 1.42 on the third day, respectively. In the present study, postoperative pain levels following cholecystectomy were highest on the first postoperative day and gradually decreased over the subsequent days. This finding is consistent with the expected course of pain reduction after surgical trauma and aligns with similar temporal trends reported in the literature (26). However, the mean postoperative pain scores observed in this study were relatively higher than those reported in some previous studies. Ilkaz et al. reported a mean postoperative pain score of 3.49 ± 1.25 following laparoscopic cholecystectomy (27), whereas Leblebici et al. reported a mean score of 4.7 ± 1.3 (28). This difference may be partly explained by the timing of pain assessment, as pain was measured at the 8th postoperative hour, prior to patient mobilization in the present study. Additionally, variability in reported pain scores across studies may be attributed to differences in the patient characteristics, anesthesia, and analgesic regimens. Therefore, these factors should be taken into account when interpreting and comparing postoperative pain outcomes.

In the present study, a higher number of postoperative step counts was associated with lower pain scores. This finding is consistent with previous studies reporting an inverse relationship between physical activity levels and postoperative pain (18, 19). Master et al., in a randomized controlled study conducted to investigate the relationship between the number of postoperative step counts and postoperative pain and opioid use in patients (*n* = 248) undergoing lumbar spine orthopedic surgery, found that the pain of the patients decreased as the number of step counts increased (18). Sharpe et al. examined the improvement of physical activity and its relationship with pain in women undergoing elective cesarean delivery in a cross-sectional study. They presented an inverse correlation between the number of step counts and pain assessments, indicating that pain decreased as the number of step counts increased (19). At the same time, many studies have reported that early postoperative mobilization and regular walking contribute positively to postoperative pain management (20, 24, 25). This relationship may be explained as bidirectional, whereby greater postoperative mobility could be associated with lower pain levels, while lower pain may, in turn, be considered to facilitate higher postoperative step counts.

Quality of recovery, another parameter addressed in the study, is recognized as a valid and important indicator in postoperative care (9, 10). In the present study, the mean quality of recovery (QoR-15) total score of cholecystectomy patients on the third day was 124.75 ± 18.71. In the literature, the mean QoR-15 scores of patients undergoing cholecystectomy range between 94 and 145 (29–31). In an experimental study conducted by Obrink et al. on women (*n* = 73) undergoing laparoscopic cholecystectomy in which a nutritious preoperative drink and chewing gum during recovery were compared with standard care, the total quality of recovery scores in the control group was 101 on the first postoperative day and 111 on the second day (29). In patients (*n* = 80) undergoing laparoscopic cholecystectomy, Han et al. found that the quality of recovery scores were 94.4 on the first postoperative day and 116.3 on the second postoperative day (32). In this study, the quality of recovery was measured on the third day and was higher compared to other studies.

In the present study, a significant association was observed between postoperative step counts and quality of recovery, as measured by the QoR-15 scale, with higher step counts being associated with higher QoR-15 scores. In the study by Ratcliffe et al., examining the effect of the number of steps on recovery in patients (*n* = 48) undergoing day surgery, the quality of recovery was followed for seven days postoperatively. The average QoR-15 total score increased from 123 on the first day to 145 on the seventh day (13). The study, which has similar findings to the current study, also states that the quality of recovery score increases as the number of postoperative steps increases. Furthermore, multivariable linear regression analysis provided additional support for the observed associations. The quality of recovery (QoR-15 total score) was independently associated with surgical approach, step count on postoperative day 3, and pain level on postoperative day 3. Specifically, higher step counts on postoperative day 3 were independently associated with higher QoR-15 scores, whereas higher pain levels on postoperative day 3 were independently associated with lower QoR-15 scores. The model suggests that postoperative mobility and pain are key factors closely related to patients' perceived recovery during the early postoperative period. Existing evidence supports a meaningful association between objectively measured physical activity and patient-reported functional recovery following surgery. In patients undergoing total hip or knee arthroplasty, individuals with higher step counts have been shown to tend to report better functional status (33, 34). Similarly, among patients with lower extremity fractures, step counts measured via smartphone-based accelerometry were moderately positively associated with physical function questionnaire scores. Moreover, threshold-based analyses propose clinically meaningful activity benchmarks; following hip fracture, achieving more than 150 steps per day at one month and more than 425 steps per day at three months has been associated with improvements in functional independence (35). Collectively, these findings suggest that objectively measured step counts serve as a valuable indicator of functional recovery trajectories. Higher postoperative step counts may be considered an essential component associated with achieving a high-quality recovery, which represents a central objective of perioperative care.

### 4.1 Strengths and limitations

The main limitation of the study is that it was conducted in a single center, and the data cannot be generalized. Additionally, the use of minimum walking distance thresholds may have introduced selection bias by excluding patients with lower preoperative functional capacity. Therefore, the findings are primarily generalizable to patients with preserved baseline mobility, and caution is warranted when extrapolating the results to more frail populations. The study focused on the quality of recovery, which is a patient's subjective data, and complication follow-up, which can be objective data, was not performed. Future studies may focus on the evaluation of objective and subjective data together.

## 5 Conclusion

This study revealed that the median number of step counts of patients who underwent cholecystectomy was 245 on the first day, 719.5 on the second day, and 983 on the third day; the mean pain level was 7.20 ± 1.10 on the first day, 5.26 ± 1.29 on the second day, 3.76 ± 1.42 on the third day; the mean score on the quality of recovery scale was 124.75 ± 18.71.

After cholecystectomy, higher postoperative step counts were found to be associated with lower pain levels and higher quality of recovery scores. Additionally, quality of recovery was independently associated with surgical approach, postoperative step count, and pain level on postoperative day 3. Many studies in the literature support these results and confirm that physical activity plays a critical role in postoperative recovery. In line with these results, it may be recommended to follow the step counts of surgical patients after early mobilization and to encourage patients to engage in physical activity. Future research may focus on studies to determine the best tool to measure physical mobility for different surgical patient groups and surgical patients.

## Data Availability

The original contributions presented in the study are included in the article/Supplementary Material, further inquiries can be directed to the corresponding author.
